# Mutations in the two ribosomal RNA genes in mitochondrial DNA among Finnish children with hearing impairment

**DOI:** 10.1186/s12881-015-0145-6

**Published:** 2015-02-04

**Authors:** Sanna Häkli, Mirja Luotonen, Martti Sorri, Kari Majamaa

**Affiliations:** Department of Otorhinolaryngology, Oulu University Hospital, Oulu, Finland; Medical Research Center, Oulu, Finland; Institute of Clinical Medicine, Department of Otorhinolaryngology, University of Oulu, Oulu, Finland; Institute of Clinical Medicine, Department of Neurology, University of Oulu, Oulu, Finland; Department of Neurology, Oulu University Hospital, Oulu, Finland; Department of Clinical Medicine, Otorhinolaryngology, University of Oulu, P.O. Box 5000, FIN-90014 Oulu, Finland

**Keywords:** Haplogroup, Hearing loss, Mitochondrial DNA, Mutations, Rare variants

## Abstract

**Background:**

Mutations in the two *MT-RNR* genes in mitochondrial DNA can cause hearing impairment that presents with variable severity and age of onset. In order to study the prevalence of mutations in *MT-RNR1* and *MT-RNR2* genes among Finnish children, we studied a ten-year cohort of hearing impaired children born in Northern Finland.

**Methods:**

We studied children, who had been born in Northern Finland in 1993–2002 and who had been ascertained to have hearing impairment by 31 December 2007. Samples from 103 children were sequenced in order to find mutations in the *MT-RNR1* and *MT-RNR2* genes.

**Results:**

One child harboured the pathogenic m.1555A > G mutation in *MT-RNR1* suggesting a frequency of 4.4/100,000 in the Finnish paediatric population. In addition, eight rare variants and 13 polymorphisms were found in *MT-RNR1* and *MT-RNR2* genes. Five of the rare variants were deemed to be haplogroup-specific polymorphisms rather than putative pathogenic mutations, while the remaining three variants have been reported in various haplogroups. Among them m.990 T > C occurs at a conserved site.

**Conclusions:**

The presence of m.990 T > C variant in various haplogroups and the rather high degree of conservation at this site suggest that this transition is a pathogenic rather than homoplasic neutral variant. Identification of further patients with m.990 T > C and segregation analysis in their families should help in determining the pathogenic potential of this variant.

## Background

Mutations in mitochondrial DNA (mtDNA) can cause hearing impairment (HI). The most prevalent of these mutations are m.3243A > G in the *MT-TL1* gene and m.1555A > G in the *MT-RNR1* gene. Among adult patients with possible matrilineal sensorineural HI, the frequency of m.3243A > G in Finland is 4.3% and that of m.1555A > G is 2.6% [1]. A population prevalence of 16-18/100,000 has been obtained for the m.3243A > G mutation by screening various patient groups in a defined population in Northern Finland [2,3]. Interestingly, the population prevalence of both m.1555A > G and m.3243A > G is approximately 1/500 in Caucasian population samples [4-6] suggesting that most subjects with the mutation remain unaffected. In addition to m.1555A > G and m.3243A > G, many other point mutations causing HI have been reported in mtDNA including those in *MT-TS1* encoding transfer RNA^Ser (UCN)^ and *MT-RNR1* encoding ribosomal 12S RNA and [7].

Mutations in *MT-RNR1* gene can cause HI with or without aminoglycoside exposure [8], whereas no mutations have been reported in the *MT-RNR2* gene in patients with HI. HI associated with mtDNA mutations can be syndromic or non-syndromic. It is most commonly sensorineural and progressive affecting mainly the high frequencies [7]. More variable patterns also occur and in these patients HI affects all frequencies, is not always progressive and can be of the conductive or mixed type [9]. Auditory neuropathy spectrum disorder (ANSD) is a clinical syndrome characterized by evidence of cochlear function in conjunction with an aberrant auditory neural system. The molecular causes of ANSD are not well known but several cases have been linked to mitochondrial disorders [10].

Specific polymorphisms and sequence variation in the D-loop define ten European mtDNA haplogroups. Mitochondrial DNA haplogroups have been shown to modulate the risk of visual failure in Leber hereditary optic neuropathy [11] and they may modulate the phenotype also in HI [12]. In addition, mtDNA haplogroups have been shown to be associated with age-related HI [13] and with hereditary HI [14].

The epidemiology of childhood HIs has previously been studied in Northern Finland in the birth cohorts of 1973–82 and 1983–92 [15]. In these studies the possible contribution of mtDNA mutations to childhood HI was not studied. In order to study the prevalence of mtDNA mutations among Finnish children with HI, we studied the next ten-year cohort of children born in Northern Finland between the years 1993–2002 and ascertained with HI before 31 December 2007. We screened 103 children with sensorineural, mild to profound, syndromic or non-syndromic HI for mutations in the *MT-RNR1* and *MT-RNR2* genes. In addition, mtDNA haplogroups were determined in order to study their contribution to childhood HI.

## Methods

### Subjects

The study population consisted of children born in Northern Finland between the years 1993–2002 and whose HI had been ascertained before 31 December 2007 (the prevalence date) at the Oulu University Hospital (OUH). OUH is responsible for the diagnostics of all HIs of children in a population of about 730,000 within an area geographically covering the northern half of Finland. Review of records at OUH revealed 240 children with HI and among them 143 children had HI with unknown aetiology. Samples were obtained from 103 unrelated children with mild to profound sensorineural HI that was deemed to be syndromic or presumably syndromic HI (N = 36) or nonsyndromic. The most common additional disability associated with HI was intellectual disability or developmental delay. In 37 children, one or more first degree relative had HI. All children with non-syndromic HI were negative for mutations in the *GJB2* gene. In addition, all children were negative for the m.3243A > G mutation and mutations in the *MT-TS1* gene. Eighteen children had received aminoglycoside antibiotics during perinatal period.

Controls consisted of 99 blood donors from the province of Northern Ostrobothnia. The donors and their mothers were required to be free of the common manifestations of mitochondrial diseases, such as diabetes mellitus, HI and neurological ailments. In addition, it was required that the donors and their mothers had been born in the same province.

The study protocol was approved by the Ethics Committee, OUH. Written informed consent was obtained from the parents.

### Molecular methods

A blood sample was obtained from the ascertained children and genomic DNA was extracted using the QIAamp Blood Kit (Qiagen, Hilden, Germany). Template DNA was first amplified in the presence of ^35^S-dATP and the amplified fragments were screened for m.1555A > G [16] by restriction fragment analysis with Alw26I (Fermentas, St. Leon-Roth, Germany). The digested samples were electrophoresed through a 6% nondenaturing polyacrylamide gel.

The *MT-RNR1* and *MT-RNR2* genes were screened for polymorphisms and mutations by using conformation sensitive gel electrophoresis (CSGE) [17] and subsequent sequencing in 88 samples or by direct sequencing in 15 samples. For CSGE, 12 pairs of primers were used to amplify the mtDNA fragments. Fragment size ranged from 202 base pairs (bp) to 397 bp. Samples forming heteroduplexes with a control strand were analyzed by automated sequencing if the heteroduplex differed in mobility on CSGE from a wild-type homoduplex. Sequencing was carried out using the BigDye Terminator v1.1 Cycle Sequencing Kit and the ABI PRISM 3130xl Genetic Analyzer (Applied Biosystems, Life Technologies Corporation, Carlsbad, CA, U.S.A.). The primers used for sequencing were the same as those used in the amplification reactions for CSGE.

The D-loop was amplified in one fragment spanning the nucleotides m.15975-m.725. The sequence was determined by use of forward primers with their 5′ nucleotides at positions m.15975 and m.16449, respectively. The sequences in some samples were also determined by use of reverse primers with the 3′ nucleotide at positions m.107 and m.457, respectively. The mean length of the D-loop sequence analyzed was 1109 bp (range, 884–1123 bp) and, on average, 98.8% of the entire fragment was covered.

A phylogenetic network based on the D-loop sequences was constructed by use of a reduced-median algorithm [18]. MtDNA haplogroups were identified on the basis of informative variants [19]. Frequencies of mtDNA haplogroups among children with HI and among the controls were compared using an exact test of population differentiation as implemented in Arlequin version 3.5.1.3. [20].

### Data analysis

MtDNA variants were identified by comparing the obtained sequences with the revised Cambridge reference sequence (Genbank NC_012920). MITOMAP database (www.mitomap.org/MITOMAP) and Human Mitochondrial database (HmtDB; www.hmtdb.uniba.it) were used to evaluate variant frequencies and to compare the frequencies in different haplogroups. A variant was defined rare if its frequency was ≤ 0.2% in either of the databases and those with frequencies > 0.2% were regarded as common polymorphisms [21].

The conservation of the nucleotides at variant sites located in the *MT-RNR-1* and *MT-RNR-2* genes was assessed. The alignments were performed by using Clustal Omega-Multiple Sequence Alignment tool (http://www.ebi.ac.uk/Tools/msa/clustalo/). For this alignment, the species and reference sequences were chosen according to the proposed consensus panel of 10 organisms [22]. It was defined that a position was rather highly conserved if nine variants were identical among the 10 organisms.

## Results

**Figure 1:**
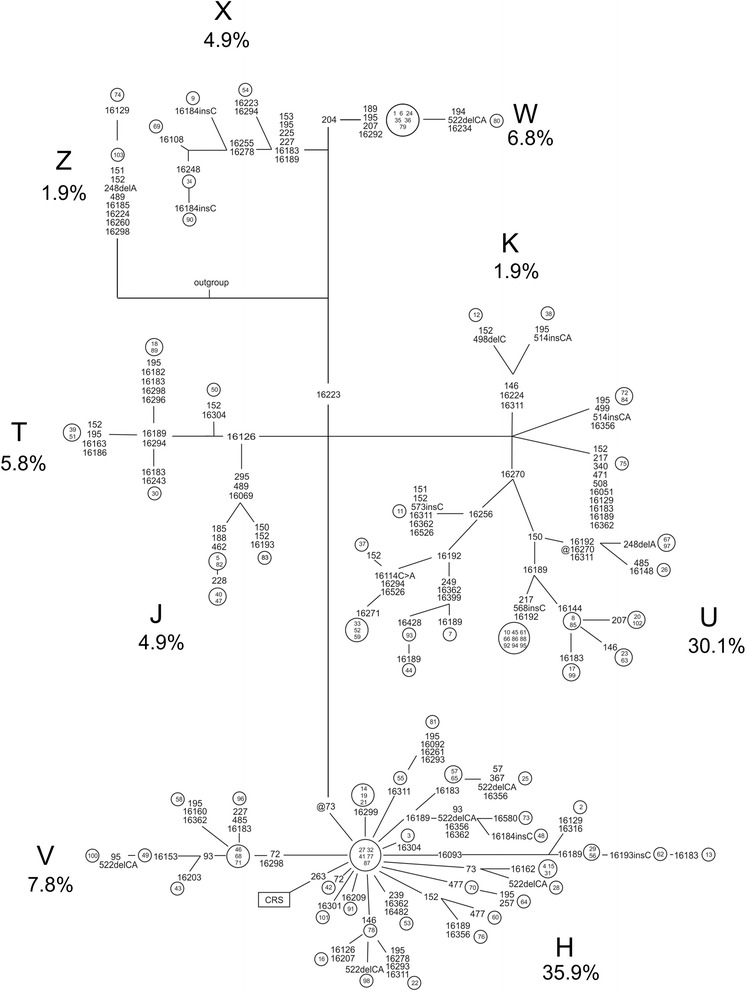
**Phylogenetic network of mtDNA.** The network was constructed on the basis of variation in the D-loop sequence of 103 Finnish children with HI. Fast-evolving sites, m.303, m.311 and m.16519 were not included in the network. The frequencies (%) of mtDNA haplogroups are shown. Numbers inside the nodes denote samples. The polymorphic variants shown on the lines connecting the nodes are transitions unless marked otherwise. ins = insertion, del = deletion, @ = back mutation. The outgroup is mtDNA from an African individual [GenBank:AF346980].

The D-loop sequences were used to infer mtDNA haplogroups and haplotypes in the 103 children with HI. The frequencies of mtDNA haplogroups did not differ from those in the general population of the province of Northern Ostrobothnia (p = 0.78, exact test of population differentiation; Figure 1). The 103 patients belonged to 66 haplotypes, 24 of which were present in the phylogenetic network of Finnish mtDNA [19], while the remaining 42 differed from the nearest neighbour by at least one substitution (Figure 1).

One child with sensorineural HI ascertained at age 4.8 years was found to harbour the m.1555A > G mutation. Her HI progressed to be severe by age 10.2 years and she received a cochlear implant at age 11.3 years. Her mother and five of her eight siblings had HI [23]. In this pedigree we were able to ascertain four children with HI, who harboured m.1555A > G and who had been born between the years 1993–2002 in Northern Finland. The 10-year birth cohort was 91,022 suggesting a minimum prevalence of 4.4/100 000 for the mutation (95% confidence interval, 1.2; 11.2). In 2007 there were 81 539 nuclear families with children in Northern Finland (Statistics Finland) suggesting that the frequency of families with a child harbouring m.1555A > G is 2.5/100 000.

Analysis of *MT-RNR1* and *MT-RNR2* sequences revealed 21 variants in addition to m.1555A > G. A variant was defined rare if its frequency was ≤ 0.2% in the Mitomap or HmtDB database and those with frequencies > 0.2% were regarded as common polymorphisms [21]. Consequently, we identified eight rare variants and 13 polymorphisms (Table 1). Among the 13 polymorphisms, five were at a frequency greater than 5%, four were at a frequency 1–5% and four were at a frequency > 0.2% and < 1% (Table 1).

**Table 1:** **Frequencies of rare variants in** ***MT-RNR1*** **(positions 648–1601) and** ***MT-RNR2*** **(positions 1671–3229) among children with HI and in database sequences**

	**Children with HI**		**HmtDB**				**Mitomap’s GenBank set**	
	**(N = 103)**		**Europeans (N = 4535)**		**All continents (N = 16,231)**		**(N = 26,851)**	
**Variant**	**(N)**	**(%)**	**(N)**	**(%)**	**(N)**	**(%)**	**(N)**	**(%)**
740G > A	1	0.97	15	0.33	22	0.14	25	0.09
896A > G	1	0.97	14	0.31	20	0.12	31	0.12
958C > T	1	0.97	1	0.02	5	0.03	8	0.03
990 T > C	1	0.97	3	0.07	11	0.07	17	0.06
1341C > T	1	0.97	5	0.11	7	0.04	18	0.07
2098G > A	1	0.97	18	0.40	33	0.20	47	0.18
2405c-cc	2	1.94	2	0.04	3	0.02	27	0.10
2445 T > C	2	1.94	4	0.09	6	0.04	9	0.03

Common polymorphisms in *MT-RNR1*, frequency in Genbank (26,851 complete or near-complete sequences in Mitomap’s GenBank Set): m.709G > A (13.1%), m.930G > A (2.1%), m.961 T > G (0.4%), m.1243 T > C (1.9%). Common polymorphisms in *MT-RNR2*: m.1719G > A (5.0%), m.1721C > T (0.7%), m.1811A > G (8.0%), m.1888G > A (5.7%), m.2259C > T (0.7%), m.2706A > G (76.1%), m.3010G > A (16.9%), m.3116C > T (0.3%), m.3197 T > C (4.4%). HmtDB: Human Mitochondrial Database, http://www.hmtdb.uniba.it.

The eight rare variants were present among the sequences in Mitomap, HmtDB or our own files and comparison of the sequences indicated that m.740G > A, m.896A > G, m.1341C > T and m.2405C-CC are strictly haplogroup-specific (Table 2). The remaining four rare variants (m.958C > T, m.990 T > C, m.2098G > A, m.2445 T > C) have been found in various haplogroups. The positions m.958 and m.2445 were not evolutionary conserved (Table 3) and children harbouring these variants belonged to haplogroups U5b and U5a that have previously been assigned to these variants. The position m.2098 was rather conserved. However, the child with m.2098G > A belonged to haplogroup H1, which was the case also in 29 out of 33 sequences in databases suggesting that m.2098G > A is a haplogroup H1 associated variant. Finally, the m.990 T > C variant occurred in subhaplogroup V2 and the position m.990 was rather highly conserved (Table 3).

**Table 2:** **Clinical features of children with hearing impairment and with rare variants in** ***MT-RNR1*** **and** ***MT-RNR2***

**Variant**	**Sex**	**Degree of SNHI**	**Other symptoms**	**Age at diagnosis (years)**	**Family history**	**Haplogroup**	**Haplogroup in HmtDB or Mitomap**
740G > A	Girl	Severe	Non-syndromic	5	Negative	Z1a	Z1a
740G > A	Boy	Moderate	Intellectual disability, short stature, renal dysplasia	10	Negative	Z1a	Z1a
896A > G	Girl	Mild	Intellectual disability cleft palate, congenital hypothyreosis	8	Negative	U5b	U5b
958C > T							U5b,M5a,M5b,M7d
2445 T > C							U5a,U5b,D1,H1,L2
990 T > C	Girl	Profound*	Non-syndromic	5	Negative	V2	L3,D4,V2,H1,H3,H4
1341C > T	Girl	Mild	Non-syndromic	11	Negative	U5b	U5b
2098G > A	Girl	Severe	Intellectual disability, hydrocephalus, spastic triplegia	3	Negative	H1	H1,K2,J2
2405c-cc	Boy	Mild	Non-syndromic	7	Negative	U4d	U4d
2405c-cc	Girl	Moderate	Non-syndromic	5	Positive dominant	U4d	U4d
2445 T > C	Boy	Severe	Non-syndromic	3	Negative	U5a	U5a,U5b,D1,H1,L2

SNHI = sensorineural hearing impairment, *= mild conductive HI on one side.

**Table 3:** **Conservation of the nucleotide positions of four rare variants in** ***MT-RNR-1*** **and** ***MT-RNR-2*** **genes**

**Taxonomic classification**	**Species**	**m.958**	**m.990**	**m.2098**	**m.2445**
Primates	Homo sapiens	C	T	G	T
	Pan troglodytes	C	T	G	C
	Pan paniscus	C	T	G	C
	Hylobates lar	C	C	G	C
Mammals	Mus musculus	A	T	G	A
	Rattus norvegicus	C	T	G	T
	Bos taurus	T	T	G	T
Vertebrates	Gallus gallus	C	T	G	C
	Gadus morhua	G	T	A	A
Invertebrates	Drosophila melanogaster	**─**	T	T	C

## Discussion

We found one child with the m.1555A > G mutation among 103 children with HI. The prevalence of m.1555A > G is generally 0.4 - 2.6% among European patients with HI [24-26], but prevalence figures higher than these have been reported among Spanish and Asian patients [27-29]. Among adult Finnish patients with possible matrilineal sensorineural HI, the frequency of m.1555A > G is 2.6% [1]. In the present study, we could estimate that the minimum frequency of m.1555A > G was 4.4/100 000 in the Finnish paediatric population and 2.5/100 000 in families with children. Population studies have suggested that m.1555A > G is common among Caucasians occurring at a frequency of about 1 in 500 [4,6]. Many mutation carriers have normal hearing levels [4,30] suggesting that the penetrance of m.1555A > G is low in the population.

Mitochondrial DNA haplogroups have been suggested to increase the risk in certain neurodegenerative diseases including age-related HI. The prevalence of age-related HI is higher in subjects belonging to haplogroups U and K [13]. Furthermore, possible excess of haplogroup cluster HV has been reported among European patients with postlingual, nonsyndromic HI [31] and the frequency of subhaplogroup D4b2 is higher in Japanese patients with HI than that in controls [14]. We did not find differences in mtDNA haplogroup frequencies between children with HI and the general population.

We attempted to detect new pathogenic mutations or rare variants that could be the cause of HI in children. We found eight rare variants, one of which, m.990 T > C, has been reported in association with HI [32]. Our patient with m.990 T > C had profound, sensorineural HI on one side and mild, conductive HI on the other side. Her mtDNA belonged to haplogroup V2, while the 17 sequences with m.990 T > C deposited in the Mitomap database belong to four haplogroups or eight subhaplogroups suggesting that this variant has emerged several times in human history. The nucleotide position m.990 is located in stem 20 of 12SrRNA and, interestingly, it is rather highly conserved [32]. These two pieces of information point to the possibility that 990 T > C is a pathogenic rather than homoplasic variant. Unfortunately, the detailed clinical features or mtDNA haplogroup data were not reported on the previous patient with HI and m.990 T > C [32].

In addition to the eight rare variants we found m.961 T > G, which has previously been reported in association with HI [33,34]. The mtDNA of our patient belonged to haplogroup H11 and this was also the case for 62 sequences in Mitomap. These data and previous considerations [35] suggest that m.961 T > G is a haplogroup-specific variant rather than a pathogenic mutation.

An increased number of rare polymorphisms have been reported among Finnish adult patients with sensorineural HI [36] and, furthermore, it has been proposed that increased sequence variation in mtDNA may be a genetic risk factor for HI. Interestingly, among the 103 children with HI we found one patient with three rare variants including m.896A > G, m.958C > T and m.2445 T > C and belonging to haplogroup U5b. This girl had a mild sensorineural HI ascertained at age 8 years. She had also congenital hypothyreosis, cleft palate and intellectual disability. A search in the HmtDB database revealed that a motif consisting of m.896A > G, m.958C > T and m.2445 T > C is found in only one sequence that is of Finnish origin [GenBank:EU784076]. The phenotype of this subject is not known and thus we cannot determine whether the motif is a rare haplogroup U5b signature or whether it contributes to syndromic HI.

## Conclusions

We found that the m.1555A > G mutation was present at a frequency of 0.97% among Finnish children with HI and our data further suggest that its frequency is 4.4/100 000 in the Finnish paediatric population or 2.5/100 000 in families with children. We detected eight rare mtDNA variants among the children with HI. Five of these variants (m.740G > A, m.896A > G, m.1341C > T, m.2098G > A, m.2405c-cc) were deemed to be haplogroup-specific polymorphisms rather than pathogenic mutations. The remaining three variants (m.958C > T, m.990 T > C, m.2445 T > C) were present in more than one haplogroup. Occurrence of a variant in different haplogroups suggests that the mutation has arisen more than once during evolution. Evolutionary conservation at such a variant site supports pathogenic potential, while non-conservation at the site suggests that the variant is a homoplasy. Hence, m.958C > T and m.2445 T > C were considered homoplasic variants, while m.990 T > C was deemed to be unclassified in terms of its pathogenic potential. Identification of further patients with m.990 T > C and segregation analysis in families with m.990 T > C should help in determining the pathogenic potential of the variant.

## Abbreviations

ANSD: Auditory neuropathy spectrum disorder
BP: Base pair
CSGE: Conformation sensitive gel electrophoresis
HI: Hearing impairment
SNHI: Sensorineural hearing impairment
HmtDB: Human mitochondrial database
mtDNA: mitochondrial DNA
OUH: Oulu University Hospital
